# Fibrosis-4 Index Is Closely Associated with Arterial Damage and Future Risk of Coronary Heart Disease in Type 2 Diabetes

**DOI:** 10.1155/2022/2760027

**Published:** 2022-07-07

**Authors:** Kentaro Watanabe, Noe Takakubo, Taro Saigusa, Akiko Nagasawa, Midori Yamana, Midori Ojima, Wataru Kameda, Shinji Susa, Kenichi Ishizawa, Hisamitsu Ishihara

**Affiliations:** ^1^Division of Diabetes and Metabolic Diseases, Department of Internal Medicine, Nihon University School of Medicine, Tokyo, Japan; ^2^Department of Neurology, Hematology, Metabolism, Endocrinology and Diabetology, Yamagata University Faculty of Medicine, Yamagata, Japan

## Abstract

This study evaluated the association between fibrosis-4 (FIB 4) index and arterial damage or future risk of coronary heart disease (CHD) in type 2 diabetes. The study subjects were 253 patients with type 2 diabetes. The FIB4 index, as a marker of hepatic fibrosis based on age, aspartate aminotransferase and alanine aminotransferase levels, and platelet count, was calculated for all subjects. Carotid intima-media thickness (IMT), carotid artery calcification (CAC), and aortic arch calcification (AAC) grade (0–2) were assessed as atherosclerotic variables. The Suita score was calculated as the future risk of coronary heart disease (CHD). We assessed whether the FIB4 index was associated with both atherosclerotic variables and the Suita score. FIB4 index was significantly associated with IMT (*r* = 0.241, *P* < 0.001) and Suita score (*r* = 0.291, *P* < 0.001). Subjects with CAC showed a significantly higher FIB4 index score compared to subjects without (1.70 ± 0.74 and 1.24 ± 0.69, respectively, *P* < 0.001), whereas the FIB4 index was significantly elevated with a higher grade of AAC (1.24 ± 0.74, 1.56 ± 0.66, and 1.79 ± 0.71, respectively, *P* < 0.001). Linear regression analysis adjusted for clinical characteristics indicated that the FIB4 index was positively associated with IMT, Suita score, CAC, and AAC grade (*β* = 0.241, *P*=0.004; *β* = 2.994, *P* < 0.001; *β* = 0.139, *P*=0.001; and *β* = 0.265, *P* < 0.001, respectively). FIB4 index is closely associated with arterial damage and future risk of CHD in type 2 diabetes.

## 1. Introduction

Subjects with the traditional risk factors of cardiovascular disease (CVD), such as dyslipidemia, hypertension, diabetes mellitus, and smoking, have a high risk of developing CVD [1]. Particularly, patients with type 2 diabetes show a high risk of future CVD development [2]. Further, subjects who show a cluster of CVD risk factors, such as metabolic syndrome (MetS) and type 2 diabetes, have a high risk of CVD development [3]. Furthermore, it was reported that nonalcoholic fatty liver disease (NAFLD) and nonalcoholic liver steatohepatitis (NASH) are closely associated with CVD mortality [4, 5], whereas NAFLD and NASH are commonly related to MetS [6], especially, patients with type 2 diabetes show a high prevalence of NAFLD [7]. Indeed, patients with NAFLD show elevated Framingham Scores [8]. Thus, it is necessary to evaluate risk assessment for developing CVD to improve the prognosis of patients with NAFLD and NASH. NAFLD shows characteristic histology on liver biopsy, including steatosis, steatohepatitis, fibrosis, and cirrhosis [9], of which hepatic fibrosis is closely associated with mortality [10]. Since the development of glycemic abnormalities is correlated with an increased risk of hepatic fibrosis in Japanese subjects [11], it is necessary to evaluate whether hepatic fibrosis is closely related to atherosclerosis and future risk of CVD development in Japanese patients with type 2 diabetes. Although several formulae using noninvasive variables exist to detect hepatic fibrosis, the fibrosis-4 (FIB4) index, which is a marker of hepatic fibrosis based on age, aspartate aminotransferase and alanine aminotransferase levels, and platelet count, is the most appropriate indicator of fibrosis in patients with NAFLD [12]. FIB4 index reflects the risk of hepatic fibrosis in type 2 diabetes. A previous study reported that the prevalence of hepatic fibrosis assessed via liver biopsy gradually increased, accompanied by an increase in the FIB 4 index in patients with type 2 diabetes who were originally diagnosed without or suspected of having hepatic fibrosis [13]. We concluded that the FIB4 index could be used to evaluate the risk of NAFLD and hepatic fibrosis in type 2 diabetes patients. Therefore, we hypothesized that the FIB4 index is associated with atherosclerosis and the risk of future CVD development.

We designed a cross-sectional study to determine the relationship between the FIB4 index and both atherosclerosis and future CVD risk in Japanese subjects with type 2 diabetes. In this study, we used carotid intima-media thickness (IMT) and aortic arterial calcification to evaluate arterial damage, and the Suita score of study subjects was calculated to estimate future coronary heart disease (CHD) risk. Carotid IMT [14] and aortic arch calcification (AAC) [15] are closely associated with future CVD risk, whereas the Suita score provides suitable risk factor categories for predicting the 10-year probability of CHD; it more accurately predicts CHD risk than the Framingham risk score in the Japanese population [16].

## 2. Materials and Methods

### 2.1. Study Subjects

Two hundred fifty-three patients with type 2 diabetes were enrolled in our study, comprised of 163 men and 90 women with an average age of 65.6 ± 10.9 years. All subjects were ambulatory and treated at either the Department of Neurology, Hematology, Metabolism, Endocrinology and Diabetology at the Yamagata University Faculty of Medicine or the Division of Diabetes and Metabolic Diseases, Department of Internal Medicine at Nihon University School of Medicine. All subjects had no history of hepatic cirrhosis, viral hepatitis, collagen diseases, including hepatic arteritis and primary biliary cirrhosis, autoimmune hepatitis, and malignant diseases. Patients with chest and cardiac diseases that affect the AAC evaluation, malignant diseases, collagen diseases, and acute and chronic inflammatory diseases and receiving immunosuppressants and steroid hormone therapy were excluded from our study.

### 2.2. Informed Consent and Ethics Regulations

The study was designed on the principles of the Declaration of Helsinki. We conducted a cross-sectional study using a clinical database. The study protocol was approved by the Ethics Committee of Yamagata University Faculty of Medicine and Nihon University School of Medicine. The protocol of this study is found on the web page of the Ethics Committee, and we provided the opportunity for participants to opt out of the study. The requirement for written informed consent was waived because of the retrospective nature of the study by our Ethics Committee.

### 2.3. Characteristics of Study Subjects

We evaluated the clinical characteristics of our study cohort, including sex, age, body mass index (BMI), smoking habit, systolic and diastolic blood pressure, hypertension, statin use, platelet (PLT) count, and biochemical variables. PLT and biochemical variables including lipid metabolism, uric acid, and HbA1c were measured after an overnight fast. PLT count was measured using a hematology analyzer, whereas low-density lipoprotein (LDL) and high-density lipoprotein (HDL) cholesterol, triglycerides, uric acid, creatinine, and HbA1c were measured using an automated analyzer. We used the estimated glomerular filtration rate (eGFR) as an indicator of renal function, which was determined by the following formula: eGFR (mL/min/1.73 m^2^) = 194 × serum creatinine^−1.094^ × age^−0.287^ × 0.739 (if female) [17]. Blood pressure was measured in a sitting position at the hospital in the morning. Patients with hypertension were defined as patients treated with hypertension or patients with blood pressure greater than 140/90 mmHg that was measured in a hospital based on the Japanese Society of Hypertension Committee for Guidelines for the Management of Hypertension [18].

### 2.4. Examination of Hepatic Fibrosis, Atherosclerosis, and Cardiovascular Risk

The FIB4 index was calculated to evaluate hepatic fibrosis in our study subjects. It is a useful variable to assess hepatic fibrosis in Japanese patients with NAFLD [19], although the FIB4 index was originally established as a simple noninvasive index to predict significant fibrosis in a patient with human immunodeficiency virus (HIV)/hepatitis C virus (HCV) coinfection [12]. The FIB4 index was determined by the following formula [19]:

(1) $$\begin{array}{c} \text{FIB}4\text{ index}=\frac{\text{age }\left(\text{years}\right)\times \text{AST}\left(\text{IU}/\text{L}\right)}{\text{PLT count}\left(10/\text{L}\right)\times \sqrt{\text{ALT}\left(IU/L\right)}}. \end{array}$$

A FIB4 index with a cutoff of 1.45 provides suitable diagnostic accuracy to predict hepatic fibrosis in the Japanese population [19].

IMT was measured as a marker to evaluate arterial injury in our study subjects. Carotid IMT is a suitable surrogate marker for future CVD risk [14]. A total of six segments of the near and far walls in the common, bifurcation, and internal carotid artery on the right and left were measured by B‐mode ultrasonography, as described in a previous study [20]. The maximum IMT, including a plaque on both sides, was defined as the IMT in all study subjects [20]. Trained physicians or ultrasonographers performed the carotid ultrasonographic measurements. IMT measurements showed variability of 8.0% in our cohort, as previously reported [20].

**Figure 1:**
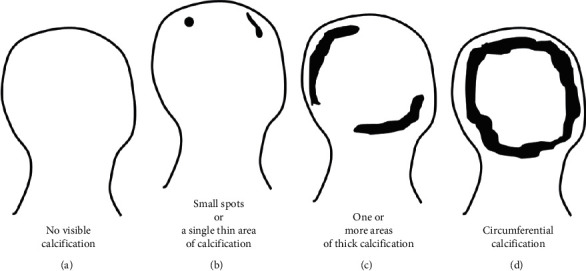
Classification of aortic arch calcification (AAC) grade. (a) grade 0, (b) grade 1, and (c, d) grade 2.

Carotid artery calcification (CAC) and the grade of AAC were evaluated as measures of arterial calcification. CAC and AAC are closely related to future CVD risk [15, 21]. The presence (*n* = 141) or absence (*n* = 112) of CAC on both sides was recorded using B-mode ultrasonography. Posteroanterior chest X-ray films were performed for study subjects, and AAC grade was reviewed in a manner as previously reported [22, 23]. Subjects were divided into three groups based on the grade of AAC as follows: grade 0, no visible calcification (*n* = 95, Figure 1(a) online); grade 1, small spots or a single thin area of calcification (*n* = 73, Figure 1(b) online); or grade 2, one or more areas of thick calcification or circumferential calcification (*n* = 83, Figures 1(c) and 1(d)). AAC assessment in this manner shows good accuracy and reproducibility [22]. In our study, one observer performed an AAC assessment. An evaluation of AAC grade showed reproducibility of 90.0% [22].

The Suita score was estimated to predict the risk of future CHD in study subjects. It is an established cardiovascular risk score using risk factor categories for predicting CHD in the Japanese population based on age, gender, smoking, diabetes, hypertension stage, LDL and HDL cholesterol, and chronic kidney disease (CKD) stage [16]. The Suita score consists of the sum of each risk score and indicates the 10-year probability of CHD [16].

### 2.5. Statistical Analysis

Pearson's coefficient correlation and univariate linear regression analysis were used to identify whether clinical characteristics, IMT, and Suita score were significantly associated with the FIB4 index. In this univariate analysis, we assumed the FIB4 index as a dependent variable and sex (men), smoking habit (current), hypertension, and statin use as independent variables. The Mann–Whitney *U* test was performed to compare the mean value of the FIB4 index in subjects between the presence and absence of CAC. Bonferroni's multiple comparison test was used to compare the mean value of the FIB4 index between AAC grades. Univariate and multivariate linear regression analysis adjusted for clinical characteristics was performed to certify whether IMT and Suita scores were significantly associated with the FIB4 index. In this univariate and multivariate analysis, the FIB4 index was assumed as an independent variable, and IMT, Suita score, CAC, and AAC grade were assumed as dependent variables. Data are presented as means ± standard deviation (SD), number (%), coefficient covariation (*r*), or *β* coefficients. Statistical significance was defined as *P* < 0.05. All analyses were performed with IBM SPSS Statistics for Windows Version 25 J (IBM Corp., Armonk, NY, USA).

## 3. Results

### 3.1. Characteristics of Study Subjects

Table 1 shows the clinical characteristics of our study cohort. A total of 163 subjects (64.4%) were men with an overall mean age of 65.6 ± 10.9 years. The number of patients with hypertension and receiving statins was 195 (77.1%) and 147 (58.1%), respectively. The mean values of systolic and diastolic blood pressure, HDL and LDL cholesterol, and triglycerides were 130.6 ± 16.8 and 76.4 ± 11.2 mmHg and 1.52 ± 0.74, 1.41 ± 0.45, and 1.55 ± 0.98 mmol/L, respectively. The mean values of BMI, eGFR, and HbA1c were 25.09 ± 4.52, 74.14 ± 25.16 mL/min/1.73 m^2^, and 7.95 ± 1.88%, respectively.

### 3.2. Relationships between FIB4 Index and Clinical Characteristics of Study Subjects

Additionally, we found that the mean FIB4 index was 1.52 ± 0.74 (Table 1), and the number of patients with no risk of hepatic fibrosis (FIB4 index ≤1.45) was 130 (51.4%; 82 men and 48 women). Diastolic blood pressure (*r* = −0.189, *P* < 0.001), eGFR (*r* = −0.243, *P* < 0.001), and HbA1c (*r* = −0.206, *P*=0.001) were significantly inversely correlated with the FIB4 index in our cohort (Table 2).

### 3.3. Relationships between FIB4 Index and Both Atherosclerosis and Future Risk of CVD

**Figure 2:**
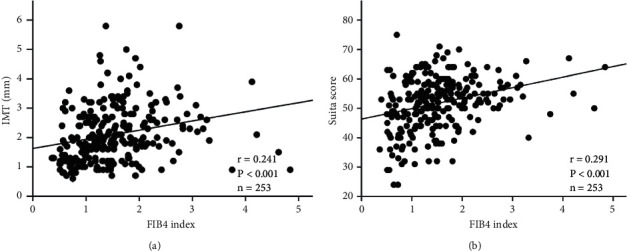
Correlations between FIB4 index and both (a) IMT and (b) suita score. FIB4 index, fibrosis-4 index; IMT, intima-media thickness.

**Figure 3:**
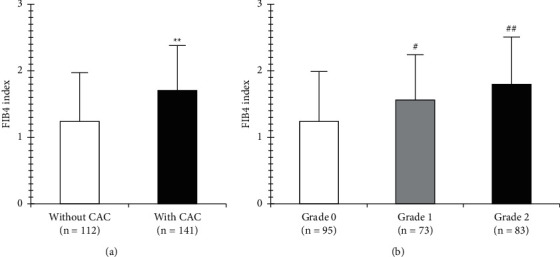
Distribution of FIB4 index in (a) CAC and (b) AAC grade. ^*∗∗*^*P* < 0.001 vs. without; ^#^*P*=0.001 and ^##^*P* < 0.001 vs. AAC grade 0. FIB4 index, fibrosis-4 index; CAC, carotid artery calcification; AAC, aortic arch calcification.

Positive correlations were found between the FIB4 index and IMT and Suita score (*r* = 0.241 and 0.291, respectively, both *P* < 0.001, Figure 2). Regarding associations between the FIB4 index and arterial calcification, we found that the FIB4 index in subjects with CAC (*n* = 141) was significantly higher than that in subjects without (*n* = 112) (1.70 ± 0.69 and 1.24 ± 0.74, respectively, *P* < 0.001, Figure 3). Further, the FIB4 index of patients with AAC grade 1 (*n* = 73) or 2 (*n* = 83) was significantly higher than that found in patients with grade 0 (*n* = 95) (*P*=0.001 and *P* < 0.001, respectively, Figure 3), although the FIB4 index increased according to a higher grade of AAC (1.24 ± 0.74, 1.56 ± 0.66, and 1.79 ± 0.71, respectively, Figure 3). Linear regression analysis indicated that the FIB4 index indicated significantly positive associations among IMT, Suita score, CAC, and AAC grade (*β* = 0.312, 3.573, 0.183, and 0.355, respectively, *P* < 0.001) (Table 3). Similarly, linear regression analysis adjusted for diastolic blood pressure, eGFR, and HbA1c indicated that the FIB4 index showed significant positive associations between IMT (*β* = 0.241, *P*=0.004), Suita score (*β* = 2.994, *P* < 0.001), CAC (*β* = 0.139, *P*=0.001), and AAC grade (*β* = 0.265, *P* < 0.001) (Table 3).

## 4. Discussion

In this study, we found that the FIB4 index was closely associated with arterial damage, such as carotid intimal thickening and arterial calcification in two major vessel beds (carotid and aortic arch). In particular, the FIB4 index was significantly increased according to the higher grade of calcification in the aortic arch. Further, we found a significantly positive correlation between the FIB4 index and the Suita score, which is a predictive marker of future CHD development. The results of this study support the new insight that noninvasive hepatic fibrosis score is significantly related to arterial damage and future risk of CHD in Japanese patients with type 2 diabetes. The clinical utility of the results of our study is that arterial damage can be easily estimated using some biochemical data (PLT count, AST, and ALT) and age in type 2 diabetes patients. Evaluation of arterial damage in patients using computed tomography, magnetic resonance imaging, carotid artery ultrasonography, pulse wave velocity, and endothelial function, among others, cannot be frequently performed. In contrast, the FIB4 index can be calculated instantly using the results of blood sampling in general practice or medical checkups. As a result, physicians can easily and frequently estimate arterial damage in patients with type 2 diabetes during general consultations or medical checkups using the FIB4 index. We conclude that the FIB4 index may be a useful tool for identifying patients with type 2 diabetes who are at high risk of CHD.

The effect of NAFLD and NASH on the future risk of CVD and mortality remains unclear. A meta-analysis study reported that cardiovascular mortality was not significantly related to NAFLD, although NAFLD increased the risk of cardiovascular events [24]. However, more severe NAFLD was significantly associated with cardiovascular mortality [24]. Indeed, some retrospective cohort studies showed that patients with NASH, but not those with simple steatosis, have a higher risk of CVD mortality [4, 10, 25]. Thus, the results of these studies suggest that hepatic fibrotic change in NAFLD and NASH is more closely related to future CVD risk and mortality than steatosis. Indeed, recent studies have shown that noninvasive scores of hepatic fibrosis were positively correlated with cardiovascular risk scores in NAFLD patients [26].

We considered some reasons why hepatic fibrosis becomes a risk of arterial damage and future CVD based on the results of past studies. First, we considered whether the worsening of glucose metabolism accompanied by the progression of hepatic fibrosis had an important role. Interestingly, overall mortality and death from CVD in patients with NAFLD without diabetes were not significantly elevated compared to those in control patients [4]. Therefore, impaired glucose metabolism may elevate the risk of CVD mortality in patients with hepatic fibrosis. A study of patients with liver cirrhosis (LC) found that insulin resistance in both the periphery (skeletal and adipose) and liver was independent of LC severity [6]. Additionally, patients with LC and diabetes showed hyperglycemia and hyperinsulinemia after a 75 g glucose load compared with patients with either normal or impaired glucose tolerance [6]. These results indicate that postprandial hyperglycemia and hyperinsulinemia induced by insulin resistance have an important role in the development of both arterial damage and CVD in patients with hepatic fibrosis. Postprandial hyperglycemia elevates arterial stiffness [27], induces carotid IMT thickening [28], and increases the risk of CVD development [29]. Hyperinsulinemia also elevates arterial stiffness [30], induces arterial intimal thickening [31], and raises CVD mortality [32].

Second, we conclude that systematic metabolic abnormalities in patients with NAFLD and NASN also contribute to the progression of arterial damage and CVD. NAFLD-induced production of pathological factors promotes multiple aspects of atherosclerosis progression. Increase of lipid particles (specifically triglycerides, very-low-density lipoprotein, and small dense LDL) [33], excessive oxidative stimuli (mainly reactive oxygen species and oxidized LDL) [34, 35], increased inflammatory factors [36], excessive hepatocytes [37, 38] and increased coagulation [39] contribute to atherosclerotic progression. These pathological factors in NAFLD promote endothelial cell damage, facilitate immune cell hyperactivation, deposition, and apoptosis, and induce vascular smooth muscle cell proliferation, migration, and differentiation, including changes in osteoblastic phenotypes [40]. We have previously shown by cross-sectional analysis that increased serum oxidative stress level was accompanied by AAC progression in type 2 diabetes [23].

Finally, we must also raise the point that MetS contributes to the progression of arterial damage and CVD in diabetes with NAFLD and NASH. NAFLD and NASH are closely associated with obesity, diabetes, hypertension, and dyslipidemia, all of which are components of MetS [41]. MetS, per se, is a risk factor for CVD development [3]. Indeed, it was reported that the progression of subclinical atherosclerosis of the coronary artery in patients with NAFLD without a history of CVD was accompanied by an increase in the number of MetS components [42].

Interestingly, we demonstrated HbA1c and diastolic blood pressure to be inversely associated with the FIB4 index. The reasons for these inverse associations remain unknown. We conclude that there may be a negative relationship between the FIB4 index and HbA1c or diastolic blood pressure with age. HbA1c and diastolic blood pressure showed significant inverse associations with age in our study subjects (*r* = −0.256 and −0.169, respectively, *P* < 0.01). Since the numerator of the FIB4 index formula contains age, the FIB4 index shows a positive association with age (*r* = 0.490, *P* < 0.001). It is reasonable to speculate that the negative associations between age and both HbA1c and diastolic blood pressure in our study account for the significant inverse association between FIB4 index and either HbA1c or diastolic blood pressure. We believe that younger subjects in our study had a more difficult time improving their glycemic control. Indeed, our study subjects who received insulin therapy were younger and had relatively high HbA1c levels (data not shown). Furthermore, diastolic blood pressure is generally decreased according to aging [43].

There were several possible limitations in our study. First, we calculated the FIB4 index in subjects including patients at low risk of NAFLD or NASH. In principle, the FIB4 index is recommended for evaluating hepatic fibrosis in patients with NAFLD/NASH, not in patients without NAFLD/NASH. We considered that the FIB4 index was linked to the risk of hepatic fibrosis because the prevalence of actual hepatic fibrosis in patients with type 2 diabetes rose in tandem with an increase in the FIB4 index underlying liver disease [13]. As a result, evidence that the FIB4 index is significantly associated with arterial damage and future risk of CHD in subgroup type 2 patients with or without NAFLD/NASH is required. However, we do not have any data on hepatic imaging, such as computed tomography, magnetic resonance imaging, or ultrasonography. Therefore, we were unable to analyze the relationship between the FIB4 index and CHD risk in the subgroups with and without NAFLD. We aim to clarify these relationships in future research. Second, study subjects may include patients with undiagnosed chronic hepatic disease, although subjects who had no history of hepatic cirrhosis, viral hepatitis, collagen diseases, including hepatic arteritis and primary biliary cirrhosis, autoimmune hepatitis, and malignant diseases were excluded. Finally, the reproducibility of the FIB4 index was not shown. We believe our study results should be validated in patients with high reproducibility in the FIB4 index at different times to increase the clinical value of our study results.

## 5. Conclusions

This study provides evidence that noninvasive assessment of hepatic fibrosis using the FIB4 index is closely associated with arterial damage and future risk of CHD in Japanese patients with type 2 diabetes. The results of our study also indicate that the FIB4 index is a suitable tool to identify those patients with diabetes at a higher risk of CVD.

## Figures and Tables

**Table 1:** Clinical characteristics of study subjects.

Clinical characteristics	Mean ± SD, *n* (%)	Range
Sex (men)	163 (64.4)	
Age (years)	65.6 ± 10.9	35–86
Body mass index	25.09 ± 4.52	14.3–40.1
Smoking habit (current)	55 (21.7)	
Systolic blood pressure (mmHg)	130.6 ± 16.8	92–181
Diastolic blood pressure (mmHg)	76.4 ± 11.2	43–105
Hypertension	195 (77.1)	
Statin use	147 (58.1)	
PLT (×10^9^/L)	231.9 ± 60.7	111–531
ALT (IU/L)	24.8 ± 14.3	7–143
AST (IU/L)	26.7 ± 21.6	7–232
FIB4 index	1.52 ± 0.74	0.36–4.84
HDL-CHOL (mmol/L)	1.41 ± 0.45	0.65–3.81
LDL-CHOL (mmol/L)	2.66 ± 0.84	0.85–8.86
Triglyceride (mmol/L)	1.55 ± 0.98	0.36–7.91
Uric acid (µmol/L)	325.5 ± 77.8	59.5–630.5
eGFR (mL/min/1.73 m^2^)	74.14 ± 25.16	14.9–176.9
HbA1c (%)	7.95 ± 1.88	5.6–18.3

SD, standard deviation; PLT, platelet; ALT, alanine aminotransferase, AST, aspartate aminotransferase; FIB4 index, fibrosis-4 index; HDL-CHOL, high-density lipoprotein cholesterol; LDL-CHOL, low-density lipoprotein cholesterol; eGFR, estimated glomerular filtration rate; HbA1c, glycosylated hemoglobin.

**Table 2:** Associations between clinical characteristics and FIB4 index.

Clinical characteristics	Correlation coefficient	*β*-coefficient (95% CI)	*P* value
Sex (men)		−0.049 (−0.242–0.143)	0.616
Body mass index	−0.097		0.122
Smoking habit (current)		−0.099 (−0.220–0.021)	0.105
Systolic blood pressure (mmHg)	0.051		0.417
Diastolic blood pressure (mmHg)	−0.189		0.003
Hypertension		0.201 (−0.017–0.419)	0.071
Statin use		0.044 (−0.143–0.231)	0.642
HDL-CHOL (mmol/L)	0.095		0.131
LDL-CHOL (mmol/L)	−0.060		0.341
Triglyceride (mmol/L)	0.001		0.990
Uric acid (*μ*mol/L)	0.052		0.413
eGFR (mL/min/1.73 m^2^)	−0.243		<0.001
HbA1c (%)	−0.206		0.001

FIB4 index, fibrosis-4 index; HDL-CHOL, high-density lipoprotein cholesterol; LDL-CHOL, low-density lipoprotein cholesterol; eGFR, estimated glomerular filtration rate; HbA1c, glycosylated hemoglobin; CI, confidence interval.

**Table 3:** Univariate or multivariate linear regression analysis predicting the association between FIB4 index, IMT, suita score, IMT calcification, and AAC grade.

Dependent variables	*β*-coefficient	95% confidence interval	*P* value
*(i) Univariate analysis*			

IMT (mm)	0.312	0.156–0.468	<0.001
Suita score	3.573	2.114–5.032	<0.001
CAC	0.183	0.103–0.263	<0.001
AAC grade	0.355	0.221–0.489	<0.001

*(ii) multivariate analysis*			

IMT (mm)	0.241	0.078–0.405	0.004
Suita score	2.994	1.662–4.326	<0.001
CAC	0.139	0.057–0.221	0001
AAC grade	0.265	0.127–0.404	<0.001

Dependent variable: IMT, suita score, CAC, and AAC grade; independent variables: FIB4 index adjusted for diastolic blood pressure, eGFR, and HbA1c. FIB4 index, fibrosis-4 index; IMT, intima-media thickness; CAC, carotid artery calcification; AAC, aortic artery calcification; eGFR, estimated glomerular filtration rate; HbA1c, glycosylated hemoglobin.

## Data Availability

The data used to support the findings of this study are available from the corresponding author upon request.
